# Intestinal Microecology of Mice Exposed to TiO_2_ Nanoparticles and Bisphenol A

**DOI:** 10.3390/foods11121696

**Published:** 2022-06-09

**Authors:** Chen Yang, Youlan Tan, Fengzhu Li, Hongbin Wang, Ying Lin, Fuping Lu, Huabing Zhao

**Affiliations:** 1 College of Biotechnology, Tianjin University of Science and Technology, 9 TEDA 13th Street, Tianjin 300457, China; chenyariapril@163.com (C.Y.); 17806284231@163.com (Y.T.); lifengzhu829@163.com (F.L.); whb@tust.edu.cn (H.W.); lfp@tust.edu.cn (F.L.); 2 Key Laboratory of Industrial Fermentation Microbiology, Ministry of Education, Tianjin University of Science and Technology, Tianjin 300450, China; 3 School of Biological Science and Engineering, South China University of Technology, Guangzhou 510006, China; feylin@scut.edu.cn

**Keywords:** TiO_2_ NPs, BPA, 16S rDNA, faecal metabolomic

## Abstract

Exposure to titanium dioxide nanoparticles (TiO_2_ NPs) and bisphenol A (BPA) is ubiquitous, especially through dietary and other environmental pathways. In the present study, adult C57BL/6J mice were exposed to TiO_2_ NPs (100 mg/kg), BPA (0, 5, and 50 mg/kg), or their binary mixtures for 13 weeks. The 16S rDNA amplification sequence analysis revealed that co-exposure to TiO_2_ NPs and BPA altered the intestinal microbiota; however, this alteration was mainly caused by TiO_2_ NPs. Faecal metabolomics analysis revealed that 28 metabolites and 3 metabolic pathways were altered in the co-exposed group. This study is the first to reveal the combined effects of TiO_2_ NPs and BPA on the mammalian gut microbial community and metabolism dynamics, which is of great value to human health. The coexistence of TiO_2_ NPs and BPA in the gut poses a potential health risk due to their interaction with the gut microbiota.

## 1. Introduction

The balance between the species within each microbial community is central to microbial homeostasis. The gut microbiome serves various functions, including energy harvesting, the neutralisation of carcinogens and drugs, and the modulation of intestinal mobility and immune responses [1,2,3]. Microbial dysbiosis can aggravate various diseases, including diabetes, obesity, Crohn’s disease, and inflammatory bowel disease [1,4,5,6]. In addition, the gut microbiota is highly sensitive to exogenous stressors, including nanoparticles (NPs) and persistent organic pollutants (POPs) [7,8,9,10].

TiO_2_ NPs, a widely used nanomaterial, are used in various areas, such as cosmetics, food, and paints. TiO_2_ is generally incorporated into many daily necessities, (e.g., candies, cheese, sauces, skim milk, and ice cream) as a food colouring agent due to its brightness and high refractive index. In addition, it is used as a flavour enhancer in off-white foods, including dry vegetables, nuts, seeds, soups, wasabi, beer, and wine. Therefore, ingestion may be an important mode of TiO_2_ exposure, and the amount of TiO_2_ exposure varies with dietary intake. Dietary exposure to TiO_2_ was estimated to be 2–3 mg/kg/day for children and approximately 1 mg/kg/day for other age groups in the United Kingdom (UK) [11]. Notably, for those who prefer sweets, the consumption of TiO_2_ can be several times higher.

Bisphenol A (BPA) is one of the most widely used industrial compounds in the world [12] and is extensively used in daily commodities, such as in plastic containers, food package materials, and food can linings. BPA molecules are linked by ester bonds that are subjected to hydrolysis when exposed to high temperatures or acidic/basic substances, leading to the release of BPA into the environment, posing a potential risk to living beings [13]. Studies have demonstrated that BPA can leach from the epoxy resins of metallic food cans [14,15,16]. Furthermore, as a representative POP and environmental obesogens, BPA is distinguished by its global dispersion, numerous routes of exposure, multiple vector species, and strong bioaccumulation [17,18].

Several studies have shown that once TiO_2_ NPs enter circulation, they cause damage to multiple tissues, such as those in the kidney, liver, brain [19], intestine [20], and lung [21]. The intestine, the longest residence organ for TiO_2_ NPs following their ingestion, is the most sensitive. Some studies have demonstrated that the long-term oral use of foods containing TiO_2_ NPs may increase the risk of colorectal cancer [7]. In vitro studies have shown that TiO_2_ NPs have antimicrobial activity and can promote the production of reactive oxygen species [22,23]. Many studies on the gut have found that they can reduce intestinal microbial diversity and short-chain fatty acid production [20,24,25]. In a manner that is similar to that of TiO_2_ NPs, BPA can alter the composition of various intestinal microbiota [8]. Studies have shown that diseases such as type 2 diabetes (T2D) and obesity are closely linked to imbalances in intestinal microecology and to the accumulation of the environmental “carcinogen” BPA [26]. Furthermore, it has been documented that interaction between TiO_2_ NPs and BPA alters their innate bioavailability and toxicity [27].

There are few studies on the effects of combined exposure to TiO_2_ NPs and BPA on the gut microbiota. These studies are limited to zebrafish and have found that co-exposure to BPA and TiO_2_ NPs changed the gut microbial community in a dose-dependent manner [28]. However, as mice are phylogenetically closer to humans and possess physiological and biochemical indicators and regulatory mechanisms that are similar to those in humans, research on mice is of greater significance. Therefore, we investigated the health effects of TiO_2_ NPs and BPA on mice.

In this study, we subchronically co-exposed C57BL/6J mice to TiO_2_ NPs and BPA. The effects of co-exposure on the structure and diversity of the mouse intestinal microflora were studied using high-throughput sequencing. The metabolic components of the mouse intestine were analysed using chromatography–mass spectrometer (GC-MS)-based on untargeted metabolomics. Finally, we studied the accumulation of contaminants and their main products and possible transformation processes in the intestine and flora.

## 2. Materials and Methods

### 2.1. Chemicals

TiO_2_ NPs and BPA were purchased from Sigma-Aldrich (St. Louis, MO, USA). The purities of TiO_2_ NPs and BPA were greater than 99.7% and 99%, respectively. BPA was initially dissolved in dimethyl sulfoxide (DMSO; purity > 99%) and olive oil. The final DMSO concentration of the solution was 0.1% (*v*/*v*).

TiO_2_ NPs were dispersed in phosphate-buffered saline (PBS) according to the exposure dose. The suspension was sonicated in an ultrasonic cell disruptor for 20 min for homogenisation. The size and shape of the TiO_2_ NPs were characterised using transmission electron microscopy (TEM, Talos G2 200X, Hillsboro, OR, USA). The size distribution and average diameter (d_TEM_) of the particles were analysed using ImageJ software (Version 1.8.0).

### 2.2. Animals and Treatment

Healthy three-week-old C57BL/6J male mice were provided by SPF (Beijing, China) Biotechnology Co., Ltd. Animal ethics approval was approved by the Animal Ethics Committee of JAK BIO company (JKX-2106-01). The mice were housed in a humidity-controlled room with a 12-h light/dark cycle and free access to water and food (AIN93G). Following a week of acclimatisation, the mice were gavaged with either TiO_2_ NPs (100 mg/kg/day) or BPA (0, 5, and 50 mg/kg/day) alone or in combination. Table 1 lists the specific groupings. After grouping, canagliflozin was administered once daily by oral gavage for 13 weeks. The animals were weighed weekly so that their gavage volumes could be adjusted. BPA was administered as a repeated gavage of 5 mg BPA/kg body weight and 50 mg BPA/kg body weight, a dose corresponding to the lowest observable adverse effect level (LOAEL) and no observed adverse effect level (NOAEL) [29]. The exposure dose of 100 mg/kg/day for the TiO_2_ NPs is equivalent to the number of sweets consumed by teenagers [30]. The mice were sacrificed by cervical dislocation after 13 weeks of exposure.

To compare the titanium (Ti) content in faeces under the combined effect of TiO_2_ NPs and BPA and under the effect of single TiO_2_ NPs, a single TiO_2_ NPs exposure model was established. The groupings are presented in Table S1.

### 2.3. Quantification of Faecal Titanium in Mice

The Ti concentration was measured in the faecal samples that were collected from the mice before sacrifice. Using a microwave-assisted reaction system, the faecal samples (100–300 mg) were weighed and put in a Teflon microwave digestion vessel along with 8 mL of 68% nitric acid and 2 mL of 30% hydrogen peroxide. After cooling, the digestion solution was transferred into a new tube and diluted to 50 mL for detection. Inductively coupled plasma mass spectrometry (ICP-MS, Thermo-Fisher Scientific Inc., Bremen, Germany) was used to determine the Ti content of the samples. Indium (20 ng/mL) was used as the internal standard. The detection limit was determined to be 0.076 ng/mL.

### 2.4. Detection of Inflammatory Cytokines and Oxidative Stress Biomarkers

The expression levels of interleukin 6 (IL-6) and interleukin 10 (IL-10) were determined using enzyme-linked immunosorbent assay (ELISA) kits for mice (Shanghai Enzyme-linked Biotechnology Co., Ltd., Shanghai, China).

Furthermore, the oxidative stress levels were assessed by measuring the in-serum levels of superoxide dismutase (SOD) and reactive oxygen species (ROS). While the SOD levels were measured using commercial kits (Nanjing Jiancheng Bioengineering Institute, Nanjing, China), for ROS measurement, ELISA kits were used.

### 2.5. 16S rDNA Sequencing and Gut Microbiota Analysis

Before the animals were sacrificed, thirty-six faecal samples were collected from the six groups. The samples were placed in 1.5 mL tubes, snap-frozen on dry ice, and kept at −80 °C. A faecal genomic DNA extraction kit was used to extract the faecal genomic DNA according to the manufacturer’s instructions (Tiangen Biotech Co., Ltd., Beijing, China). The Illumina HiSeq platform was used to analyse the 16S ribosomal RNA genes (16S rDNA) in the faecal samples (Shenzhen HuaDa Gene Co., Ltd., Shenzhen, China). Reactions were performed in triplicate, and the genomic DNA was amplified using bacterial 16S RNA gene (V3-V4 regions)-specific primers: 341F (5′-ACTCCTACGGGAGGCAGCAG-3′) and 806R (5′-GGACTACHVGGGTWTCTAAT-3′). The QIIME (Version 1.8.0) and R (Version 4.0.5) software packages were used to analyse the data obtained.

### 2.6. Measurement of Short-Chain Fatty Acids (SCFAs)

The SCFAs in the caecal contents, including acetic acid (AA), propionic acid (PA), isobutyric acid (IBA), butyric acid (BA), isovaleric acid (IVA), and hexanoic acid (HA), were assayed through gas chromatography–mass spectrometry (GC-MS, Agilent, Santa Clara, CA, USA). Caecal contents (100 mg) were suspended in 100 μL of a 20% phosphoric acid solution and adequately homogenised for 2 min using a vortex mixer. The suspension was centrifuged at 18,000× *g* for 10 min. The supernatant resulting from the centrifugation was extracted with 500 μL of ethyl acetate followed by centrifugation at 14,000× *g* for 10 min. A 500 μM amount of 4-Methylvaleric acid was added as the internal standard. The parameters for GC-MS analysis are presented in Table S2 in the Supplementary Information. The Agilent Mass Hunter software was used to process the data.

### 2.7. Metabolomic Analysis

(1) The homogenisation of faecal matter and sample preparation

Approximately 40 mg of each sample was weighed and added to 0.5 mL of pre-cooled MeOH/H_2_O (8/2, *v*/*v*) buffer containing 0.6 μg/mL ribitol as an internal standard. The faeces were homogenised using a tissue homogeniser and then centrifuged at 13,500 rpm for 15 min at 4 °C. The supernatant (300 μL) was collected and dried under a nitrogen stream. Subsequently, 50 μL of freshly prepared methoxyamine hydrochloride (20 mg/mL pyridine) was added to the dried sample, and the mixture was incubated at 60 °C for 30 min. Next, 30 μL of N-Methyl-N-(trimethylsilyl) trifluoroacetamide with 1% trimethylchlorosilane (MSTFA + 1%TMCS) and 30 μL pyridine was added, and the sample was further incubated at 60 °C for 30 min.

(2) Analysis of non-targeted metabolomics

Detection was performed using a GC-MS instrument (5977 B, Agilent, USA). The parameters for GC/MS analysis are shown in Table S3 in the Supplementary Information, and Mass Profiler Professional (MPP) was used for data normalisation and statistical analysis. A total of 189 metabolites were identified and integrated from the GC/MS analysis of the faecal samples.

### 2.8. Histology

The animals were sacrificed after 13 weeks. Colons were fixed in 4% paraformaldehyde for 24 h and then processed for paraffin embedding, sectioning, and haematoxylin and eosin (H&E) staining, as previously described [25].

### 2.9. Statistical Analysis

SPSS 26.0 software (IBM SPSS Inc., Chicago, IL, USA) was used to analyse the data and to provide a randomization schedule. One-way analysis of variance (ANOVA) was used to assess the differences in the mean values of various parameters, and the Waller–Duncan test was used as a post doc test. After normalising the sample (sum normalisation) and scaling the data (auto-scaling) using MetaboAnalyst 5.0 [31], PLS-DA, heatmap, and KEGG pathway analyses were performed. The Spearman correlations between the gut metabolites and microbiota at the genus level were determined, and heatmaps were generated using the psych package (https://CRAN.R-project.org/package=psych (accessed on 20 September 2021)) in R (Version 4.0.5). The level of statistical significance for all tests was set at *p* < 0.05.

## 3. Results

### 3.1. Physicochemical Properties of TiO_2_ NPs

**Figure 1:**
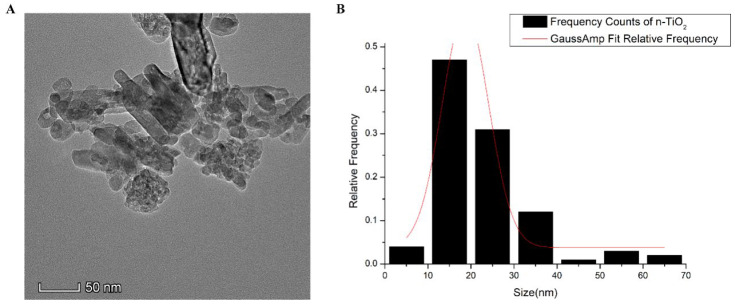
(**A**) Morphology of TiO_2_ NPs observed by TEM; (**B**) size distribution of TiO_2_ NPs measured by TEM.

The majority of the TiO_2_ NPs used in this study were cuboidal and anatase crystals with a purity of 99.90%. As shown in Figure 1, the size of most of the TiO_2_ NPs measured by TEM was 10–30 nm.

### 3.2. Effects of Coexposure of TiO_2_ NPs and BPA on the Diversity of Gut Microbiota

After 16S rDNA gene sequencing and quality filtering, 279.05 million paired reads were obtained, corresponding to a mean of 7.75 ± 2.5 thousand paired reads per sample. A total of 1412 operational taxonomic units (OTUs) were obtained from the six groups. The species accumulation curve (Figure S1) for all of the samples supports the adequacy of the sampling efforts.

**Figure 2:**
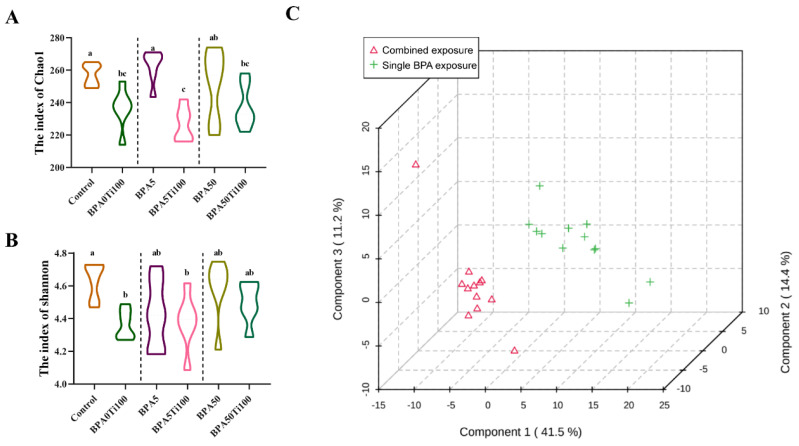
α- and β-diversity of intestinal microbiota. (**A**) Chao1, (**B**) Shannon, and (**C**) PCA score plots of the combined exposure groups (BPA5Ti100 and BPA50Ti100) and the single BPA exposure groups (BPA5 and BPA50). The same letters represent no significant differences among groups (*p* > 0.05).

To evaluate differences in the composition of the gut microbiota, α− and β−diversity analyses were performed. Compared to the BPA-only exposure groups, the Chao1 (Figure 2A) and Shannon (Figure 2B) indexes were all reduced in the combined group, suggesting that the co-exposure of BPA and TiO_2_ NPs can significantly reduce the diversity of the gut microbiota and that TiO_2_ NPs may play a dominant role in the combined effect. The β-diversity analysis was performed by dividing the groups into two groups according to BPA-only exposure and co-exposure to TiO_2_ NPs and BPA, and it revealed a clear separation between the two groups (Figure 2C). These results suggest that the diversity of mouse intestinal flora may be strongly influenced by the combined effects of TiO_2_ NPs and BPA.

### 3.3. Effects of Coexposure of TiO_2_ NPs and BPA on the Composition of Gut Microbiota

**Figure 3:**
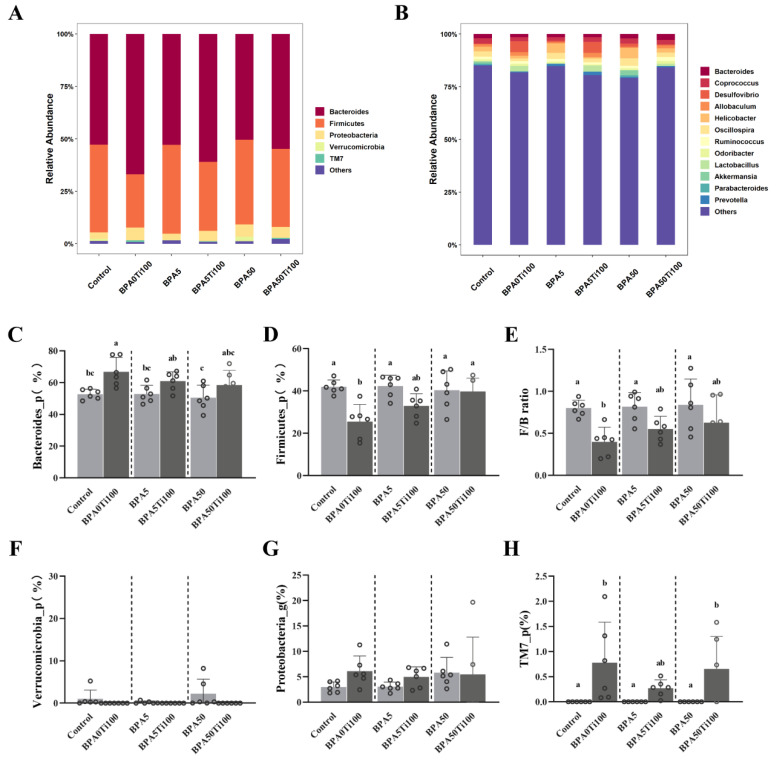
Compositional changes in the microbiota at the (**A**) phylum level and (**B**) genus level. (**C**–**H**) The relative abundance of faecal microbiota at the phylum level. Data represent the means of the relative abundance of bacteria taxa ± SEM. The same letters represent no significant differences among groups (*p* > 0.05).

Bacteroidetes, Firmicutes, Proteobacteria, Verrucomicrobia, and TM7 were dominant at the phylum level (Figure 3A,C−H). Compared to the control group, the single BPA exposure groups did not experience significant alterations in the dominant bacteria at the phylum level, whereas the BPA0Ti100 group demonstrated a greater impact on Firmicutes and Bacteroidetes, with a 39% decrease in Firmicutes and a 27% increase in Bacteroidetes (*p* < 0.05) (Figure 3C,D). However, the effect of the combined exposure on the relative abundance of Bacteroidetes and Firmicutes gradually declined as the dose of BPA increased. As a consequence, the effect of combined exposure to BPA and TiO_2_ NPs on the abundance of major species in the intestine was not significant: a plausible explanation for this phenomenon is that BPA antagonised the effect of the TiO_2_ NPs.

The relative abundance of Bacteroidetes did not differ significantly among the co-exposure groups. However, as the BPA concentration increased, the relative abundance of Firmicutes in the co-exposure groups also increased, with a significant difference observed between the BPA0Ti100 and BPA50Ti100 groups (*p* < 0.01). The ratio of Firmicutes and Bacteroidetes (F/B) altered when the relative quantity of Firmicutes fluctuated. Previous studies have reported that the ratio of Firmicutes to Bacteroidetes is strongly associated with various metabolic diseases such as obesity [32]. The F/B ratio of the BPA0Ti100 group was approximately 50% lower than that of the control group. However, as the concentration of BPA increased, the F/B ratio in the co-exposure groups increased, indicating that BPA antagonised the effect of TiO_2_ NPs on the F/B ratio.

In addition, we observed an abnormal increase in the relative abundance of the inflammatory bowel disease (IBD)-related bacteria TM7 in the group supplemented with TiO_2_ NPs [33]. A previous study indicated that IBD can be aggravated by TiO_2_ NPs, which corresponds to our results [34].

At the genus level, the combination of TiO_2_ NPs and BPA affected the composition of the gut microbial community, altering the populations of *Lactobacillus*, *Oscillospira*, and *Odoribacter* (Figure 3B and Figure S2). The relative abundance of *Lactobacillus* was almost zero in the BPA-only group but significantly increased in the combined group receiving both TiO_2_ NPs and BPA. The abundance of *Oscillospira* was found to be significantly lower in the BPA50Ti100 group than it was in the BPA50 group (*p* < 0.05). The abundance of *Odoribacter* decreased by 52.7% in the BPA5Ti100 group compared to in the BPA5 group. The BPA50Ti100 group showed a 47.4% increase over the BPA50 group. In conclusion, it can be stated that TiO_2_ NPs strongly influence the relative abundance of inflammation-related intestinal microbes.

### 3.4. Effects of Coexposure of TiO_2_ NPs and BPA on the Production of Short-Chain Fatty Acid

SCFAs are significant microbial degradation metabolites that play vital roles in host health. Acetic acid, propionic acid, and butyric acid are the main components of SCFAs, accounting for 90–95% of the total amount of SCFAs.

**Figure 4:**
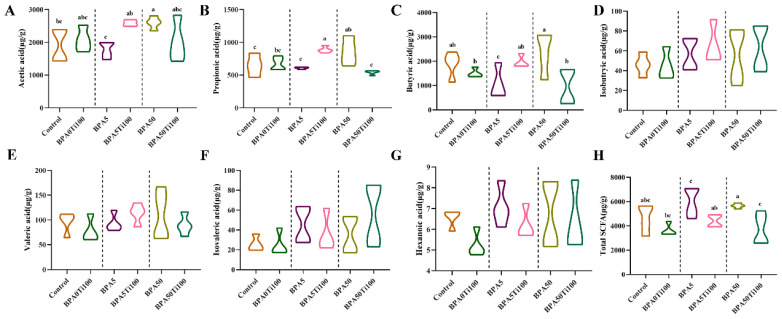
The levels of (**A**) Acetic acid; (**B**) Propionic acid; (**C**) Butyric acid; (**D**) Isobutyric acid; (**E**) Valeric acid; (**F**) Isovaleric acid; (**G**) Hexanoic acid and (**H**) Total SCFA. The same letters represent no significant differences among groups (*p* > 0.05).

Compared to the control group, the low-dose BPA-only group showed a decrease in acetic acid, propionic acid, and butyric acid (Figure 4A–C). On the other hand, the high-dose BPA-only group showed the opposite result. Compared to the corresponding BPA-only group, the levels of acetic acid, propionic acid, and butyric acid increased in the BPA5Ti100 group, whereas there was a decrease in the levels of propionic acid and butyric acid in the BPA50Ti100 group, which may represent the biological effect of BPA being antagonised by TiO_2_ NPs.

In terms of total SCFAs, our results revealed that the combined effect of TiO_2_ NPs and BPA considerably lowered SCFA levels in the caecum compared to each BPA-only group (Figure 4H). The TiO_2_ NPs effectively reduced the total number of SCFAs, which was mainly due to the reduction in propionic acid and butyric acid. The total SCFAs remained somewhat steady in the composite group compared to the group BPA0Ti100 group, which was most likely because the TiO_2_ NPs antagonised the biological impact of BPA.

### 3.5. Effects of Coexposure of TiO_2_ NPs and BPA on the Inflammatory Response

**Figure 5:**
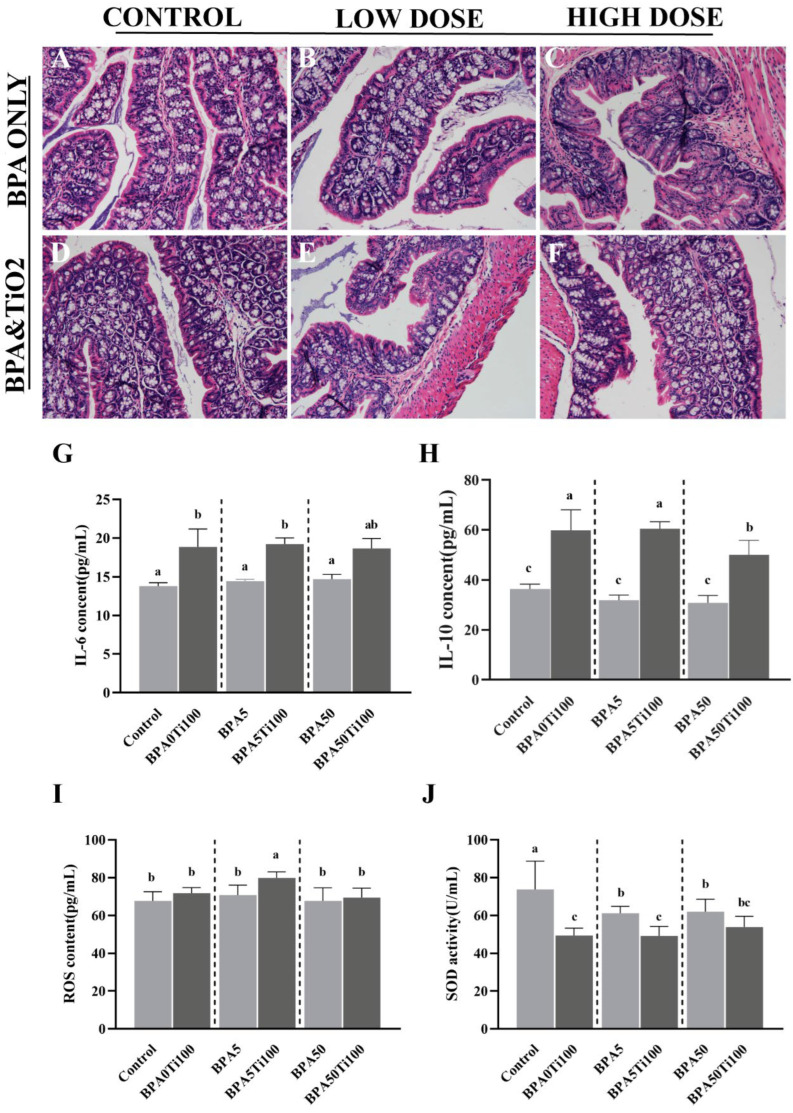
Inflammatory response of TiO_2_ NPs and BPA. (**A**–**F**) Histological characterisation (H&E staining) of the colonic tissue of mice (200×); (**G**) in-serum IL-6 levels; (**H**) in-serum IL-10 levels; (**I**) in-serum ROS levels; (**J**) in-serum SOD levels. The same letters represent no significant differences among groups (*p* > 0.05).

Our data indicate that the combination of TiO_2_ NPs and BPA causes intestinal microbial dysregulation in mice, in which TiO_2_ NPs play a critical role in microbial dysbiosis. Previously, TiO_2_ NP-induced alterations in the gut microbiota have been reported to be associated with IBD [7]. Therefore, we hypothesised that the gut microbial dysbiosis caused by TiO_2_ NPs and BPA might be related to IBD. Figure 5A–F show the changes in the colon morphology of mice induced by oral exposure to TiO_2_ NPs and BPA for 13 weeks. At various doses of BPA-only exposure, no significant differences were observed in the H&E staining results compared to the control group, while the BPA50Ti100 group showed a moderate distortion of the crypt structure and moderate goblet cell loss.

Additionally, we measured the concentrations of the pro-inflammatory cytokine IL-6 (Figure 5G) and anti-inflammatory cytokine IL-10 in the serum (Figure 5H). The IL-6 and IL-10 levels were elevated in all of the combined exposure groups, with significant differences being observed in the IL-6 and IL-10 levels in the BPA0Ti100 and BPA5Ti100 groups compared to those observed in the BPA-only group (*p* < 0.05). Considering that the progression and pathogenesis of IBD are associated with oxidative stress and irregularly elevated ROS levels [35], we examined the ROS (Figure 5I) and SOD levels (Figure 5J). We found that the ROS content in the serum was significantly higher in the BPA5Ti100 group than it was in the other groups (*p* < 0.05). SOD plays a vital role in reducing ROS production. In this study, the SOD levels in the BPA0Ti100 group were found to be similar to those in the BPA5Ti100 and BPA50Ti100 groups. Therefore, it is reasonable to infer that TiO_2_ NPs are an important contributor to SOD reduction.

### 3.6. Effects of Coexposure of TiO_2_ NPs and BPA on the Gut-Associated Metabolism

**Figure 6:**
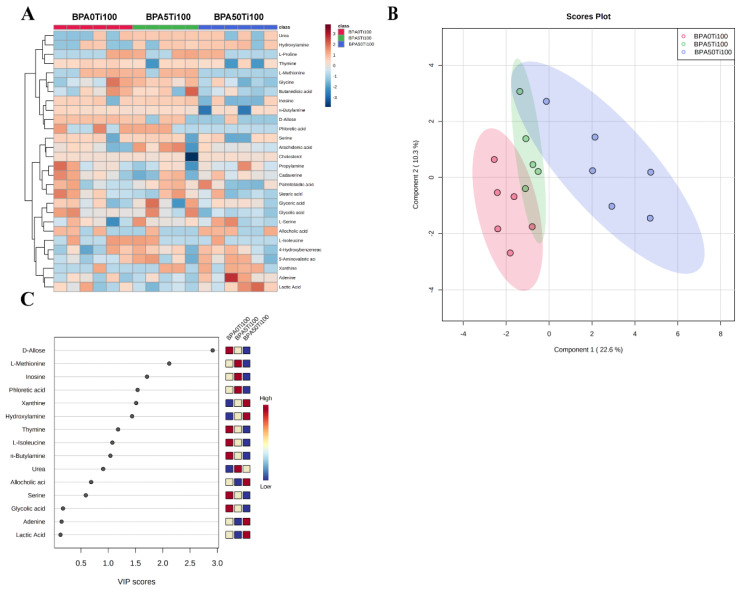
Important discriminatory metabolites were identified by clustering, correlation, and multivariate analysis between the BPA0Ti100, BPA5Ti100, and BPA50Ti100 groups. (**A**) Hierarchical clustering analysis (HCA) for the metabolites in the three groups based on their z-normalised abundances. (**B**) PLS-DA 2D score plot displaying the grouped discrimination of the Ti, 5 Ti, and 50 Ti groups by the first two P.C.s. (**C**) Variable importance in projection (VIP) scores of the important discriminatory metabolites obtained from the PLS-DA models.

The metabolomic analysis yielded 189 different metabolic features defined by their retention time and mass/charge. One sample was removed because of a technical failure. The interference effect of exposure to TiO_2_ NPs and BPA (BPA0Ti100, BPA5Ti100, and BPA50Ti100) on the metabolites was elucidated through a heatmap based on the metabolite peak area ratios, which were calculated by dividing the metabolite peak areas by the internal standard peak areas (Figure 6A). After adjusting for multiple comparisons, 28 metabolites were found to be significantly altered.

The PLS-DA 2D scores clearly showed the differences between the BPA0Ti100, BPA5Ti100, and BPA50Ti100 groups based on the first two principal components, component1 (22.6%) and component2 (10.3%), suggesting BPA5Ti100 and BPA50Ti100 group-specific metabolomic abundance and signatures (Figure 6B). The PLS-DA VIP score plot showed that several specific metabolites such as D-Allose, L-Methionine, inosine, phloretic acid, xanthine, and hydroxylamine were able to differentiate between the mice from the BPA0Ti100, BPA5Ti100, and BPA50Ti100 groups (Figure 6C). These metabolites are intimately connected to amino acids, carbohydrates, and purine metabolism. Our data strongly suggest that in the combined groups, the faecal metabolites varied with the BPA dose.

**Figure 7:**
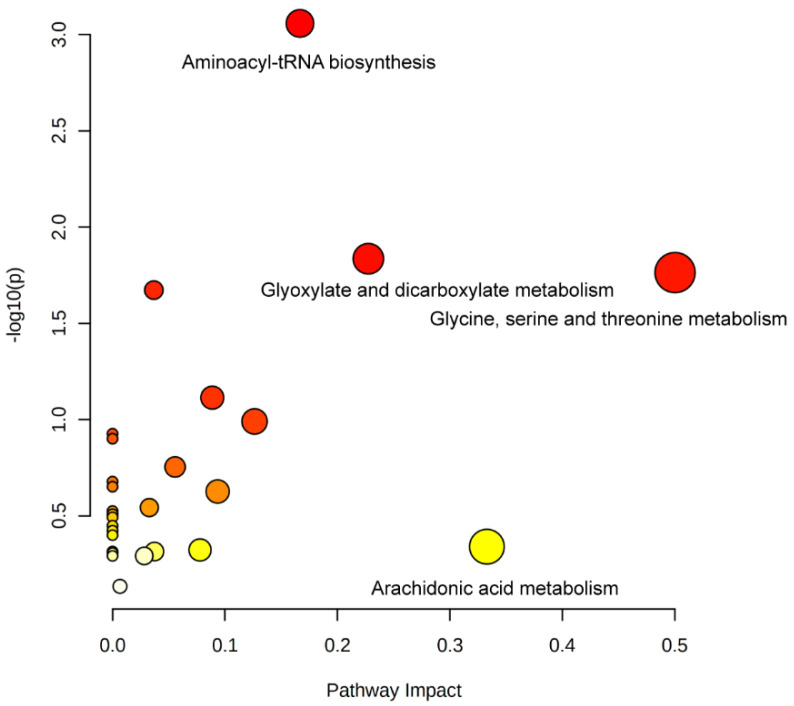
KEGG pathway analysis of differential metabolites between the samples in the BPA0Ti100 and BPA5Ti100 groups. X-Axis represents the Pathway Impact obtained by the out-degree centrality algorithm. The size of the point is related to the Pathway Impact. Y-Axis represents the negative logarithm of the p-value (−log(p)) obtained by the pathway enrichment analysis. The yellow-red color change of the point is positively related to the −log(p). The names of pathways are labeled in the graph with−log(p) > 1 or pathway impact > 0.1.

Figure 7 shows the KEGG pathway analysis of differential metabolites between the samples in the BPA0Ti100 group and BPA50Ti100-treated group. The *X*-axis represents the pathway impact obtained by the out-degree centrality algorithm. The size of the point is related to the pathway impact. The *Y*-axis represents the negative logarithm of the *p*-value (−log(p)) obtained by the pathway enrichment analysis. The yellow and red colour change of the point is positively related to the −log(p). The names of the pathways are labelled in the graph with −log(p) > 1 or with a pathway impact > 0.1.

The KEGG database was used to undertake pathway analysis of differential metabolites, revealing the effects of co-exposure of two pollutants. When we compared the BPA0Ti100 and BPA5Ti100 groups, no significant changes in the pathway were observed. The BPA5Ti100 group was compared to the BPA50Ti100 group, and it was found that the pathways remained the same as those in the BPA0Ti100 and BPA50Ti100 groups (Figure 7). Among them, aminoacyl-tRNA biosynthesis; glyoxylate and dicarboxylate metabolism; glycine, serine, and threonine metabolism were significantly altered in the group that was co-exposed to BPA and the TiO_2_ NPs. The results showed that pathways related to amino acid metabolism, genetic information processing, and carbohydrate metabolism were significantly enriched.

### 3.7. The Relationship between Faecal Metabolites and Gut Microbiota

**Figure 8:**
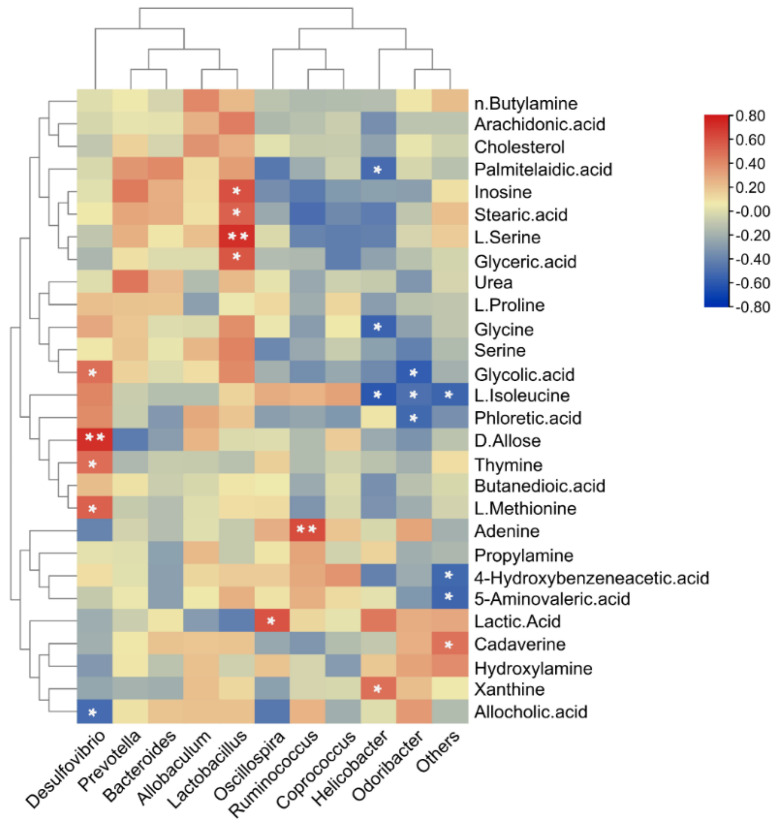
Spearman correlation heatmap analysis between gut microbiota and significant changes in faecal metabolites. *p*-values computed by Spearman correlation tests (* *p* < 0.05, ** *p* < 0.01).

Correlations between microbes and metabolites arose due to either the catabolism/anabolism of the metabolites by microbes or via the stimulation/inhibition of microbial growth by metabolites. We computed Spearman’s correlation coefficients for the metabolites and microbiome variables to study the potential dependencies between the microbiome composition and faecal metabolism (Figure 8). The results showed that most of the altered bacteria were significantly correlated with a series of differential metabolites (r > ±0.5, *p* < 0.05). These bacteria mainly belonged to the Firmicutes (*Ruminococcus*, *Oscillospira*, *Lactobacillus*), Proteobacteria (*Helicobacter*, *Desulfovibrio*), and Bacteroidetes (*Odoribacter*) phyla. The significantly correlated metabolites were assigned to lipid metabolism (stearic acid), amino acid metabolism (l-serine, glyceric acid, glycine, l-isoleucine, l-methionine, 5-Aminovaleric acid, cadaverine), carbohydrate metabolism (glyceric acid, d-allose, and lactic acid), nucleotide metabolism (inosine, thymine, adenine, xanthine), biosynthesis of other secondary metabolites (glycolic acid, allocholic acid), and metabolites without metabolic pathways (palmitelaidic acid, phloretic acid, and 4-Hydroxybenzeneacetic acid). Our study demonstrated that the co-exposure of mice to TiO_2_ NPs and BPA disturbed both the gut microbiome and faecal metabolome, and the altered gut microbiota affected metabolism.

## 4. Discussion

BPA is an endocrine disruptor that has the potential to mimic oestrogen and can thereby exert harmful health effects [36]. The extensive use of polycarbonate plastics and epoxy resins in consumer items has led to widespread BPA exposure in humans, with BPA identified in the urine of 92.6% of the American population [37]. Previous studies have shown that a single exposure to TiO_2_ NPs or BPA disrupts the metabolic balance, intestinal microbiota, intestinal oxidative stress, and inflammatory reactions [38,39,40,41]. When TiO_2_ NPs are utilised as medication carriers in the human body or when people consume the food containing E171 (36% of which is constituted by TiO_2_ NPs) [42], interactions between TiO_2_ NPs and BPA may occur [43]. Previous studies have noted that TiO_2_ NPs could enhance the bioavailability and toxicity of co-existing toxicants in the aquatic phase. The simultaneous presence of BPA and TiO_2_ NPs causes neurotoxicity [27], reproductive toxicity [44], and disturbances in the intestinal microecology of zebrafish [28]. However, the effects of the simultaneous oral exposure of mammals to BPA and TiO_2_ NPs remain unclear. In this work, we evaluated the subchronic and combined impacts of TiO_2_ NPs and BPA on the intestinal microbiota dynamics in C57BL/6J mouse models and analysed faecal metabolites to decipher the association between the two. The study revealed that combined exposure to TiO_2_ NPs and BPA altered the composition of the gut microbial community, disturbed the synthesis of metabolites such as SCFAs, and induced intestinal damage and inflammation.

Toxicological research on the effects of TiO_2_ NPs and BPA on the gut microbiota is limited. An environmental toxicology study found that combined exposure to BPA and TiO_2_ NPs shifted the intestinal microbiota in zebrafish in an antagonistic manner when the BPA concentration was low but in a synergistic manner at a higher BPA concentration [28]. We did not observe this phenomenon in this study. This could be attributed to differences in the doses of BPA and TiO_2_ NPs, routes of administration, and model animals. However, we did detect some major alterations in the gut microbiota. Compared to the combined group, the changes in the microbiota in the BPA-only group were minor. The alterations in the microbiota were significant, even in the BPA0Ti100 group. Evidently, the TiO_2_ NPs and BPA worked together to influence the gut microbial community, and the TiO_2_ NPs were mostly responsible for the alterations in the gut bacteria that were observed in the combined exposure group. The relative abundance of *Lactobacillus* was significantly higher in the co-exposure group compared to in the BPA-only group, with significant differences being observed between the BPA5 and corresponding BPA5Ti100 groups (*p* < 0.05). An in vitro study showed that the coexistence of *Lactobacillus* with nanoparticles weakened the nano-effects by controlling enzyme production [45]. Additionally, *Lactobacillus_gasseri* has been discovered to be a cause of Fournier’s gangrene and can produce ROS such as H_2_O_2_ [46]. It is possible that the greater abundance of *Lactobacillus* in the BPA5Ti100 group led to an increase in the ROS levels in this study. In the combined TiO_2_ NPs and BPA exposure groups, both *Oscillospira* and *Odoribacter* were reduced to various degrees. The BPA5Ti100 group had a much lower abundance of *Odoribacter* than the other groups did.

Similarly, research on diethylhexyl phthalate and BPA-only exposure revealed a decrease in *Odoribacter* [39,47]. Both *Oscillospira* and *Odoribacter* are producers of butyrate in the intestine [48,49]. Notably, changes in the relative abundance were not dose-dependent regarding the BPA content in the combined exposure group. This could be due to two causes. On the one hand, the toxic effects of BPA as an endocrine disruptor are more potent at low doses than at high doses within a certain range [50]. This effect even persisted when the TiO_2_ NPs were used. BPA and TiO_2_ NPs may be mutually synergistic in the intestine. As shown in the previous results for faecal Ti content (Figure S3), the Ti content in mice in the BPA5Ti100 group was slightly lower than it was in the BPA50Ti100 group. In other words, the amount of Ti that remained in the mice was slightly increased. The interaction between the TiO_2_ NPs and BPA in the gut is thought to result in increased toxicity at low BPA concentrations.

However, one limitation of this study was the use of 16S sequencing. Some bacteria could not be identified to the species level on the basis of 16S rDNA gene sequence analysis, due to minimal sequence diversity. Further investigations combined with other examination approaches should be conducted to give a clearer picture of the microbial community alternation.

Changes in the gut microbiota can send signals through the gut and produce bacterial metabolites that alter metabolism at different levels. Therefore, we performed a metabolomic analysis of the obtained mouse faeces to further explore the metabolic changes associated with the intestine [40]. Bioinformatics analysis of the faecal metabolome revealed that the BPA5Ti100 group had the greatest changes in metabolomic characteristics when compared to the BPA0Ti100 group, whereas only a few metabolites showed significant changes in the BPA50Ti100 group. This may also be due to the low dose impact indicated earlier.

In the intestine, proteins are initially broken down into peptides or amino acids by proteases that are released by the intestinal flora. Amino acid metabolism is tightly tied to gut bacteria and is altered when the intestinal flora is disrupted. Several amino acids, including L-methionine and L-isoleucine, were shown to have varied abundances in distinct metabolites. Differences in thymine, inosine, adenine, and xanthine expression suggest intestinal purine and pyrimidine metabolism issues. Allocholic acid is a bile acid that aids fat and sterol excretion, absorption, and transport. Lactic acid can be produced in the intestine by certain microbes, (e.g., *streptococci*). Although lactic acid makers can ferment carbohydrates to produce lactic acid, lactic acid accumulation lowers the intestinal pH, irritates the intestine, and increases inflammation in disease situations [51].

The metabolites were mapped to the KEGG metabolic pathway database to better understand the groups to which the differential metabolites belong. According to the KEGG enrichment study, the metabolic pathways generated by the combined action of BPA and TiO_2_ NPs differed significantly. The significantly altered metabolites were mostly implicated in the anabolic pathways for aminoacyl-tRNA synthesis; glyoxylate and dicarboxylic acid metabolism as well as in the glycine, tryptophan, and serine metabolic pathways. The differential metabolism of these amino acids and aromatic amino acids can reduce the risk of T2DM and obesity in individuals and are favourably related to insulin resistance and hyperglycaemia [52,53]. The aminoacyl tRNA synthesis pathway involves a variety of amino acid metabolic pathways, including glycine, tryptophan, and serine metabolism, which may affect the translation process by influencing the tRNA function of these amino acids. Chen et al. reported that the intake of TiO_2_ NPs affected the aminyl tRNA synthesis pathway in rat intestines [40]. Therefore, we hypothesised that this abnormality in the metabolism under the combined effect of the two contaminants was caused by TiO_2_ NPs. In terms of energy supply, glyoxylate and dicarboxylic acid metabolism are a TCA cycle back-supplementation pathway that provides the TCA cycle with an efficient energy-producing function. Abnormal glyoxylate and dicarboxylic acid metabolism indicate an abnormal energy supply to the intestinal flora. Spearman’s correlation analysis revealed a significant association between the genus-level gut microbiota and faecal metabolites. These findings imply that disruptions in the gut microbiota may result in significant changes in lipid, amino acid, carbohydrate, and nucleotide metabolism.

According to our results, the synergism between the TiO_2_ NPs and BPA was the main cause of the altered intestinal microbiota and faecal metabolites. The TiO_2_ NPs caused the greatest disruption to the intestinal bacteria, but when combined with BPA, they also exhibited aberrant changes in intestinal microbes such as *Odoribacter*. The alterations mentioned above were most likely caused by the unique adsorption behaviour of the organic pollutants BPA and TiO_2_ NPs, which influence the release of complex kinetics and alter their bioavailability, bioaccumulation, and toxicity under combined exposure.

## 5. Conclusions

In this study, we investigated the effects of subchronic exposure to TiO_2_ NPs and BPA on the intestinal microbiota of mice. We discovered noteworthy correlations between the intestinal metabolites and the gut microbiota in mice that had been co-exposed to TiO_2_ NPs and BPA, and a range of altered metabolites was detected. However, the interaction between BPA and TiO_2_ NPs is complex and cannot be simply described as being synergistic or antagonistic. More studies are needed to elucidate the mechanism of action between the two. Furthermore, the health risks that are associated with dietary exposure to TiO_2_ NPs and BPA should be noted.

## Figures and Tables

**Table 1:** Animal grouping and treatments.

Groups, *n* = 6	Treatment	Gavage Dose and Duration (13 Weeks)
Control	Olive Oil + DMSO (Vehicle)	10 mL/kg/day
BPA0Ti100	TiO_2_ NPs + Olive Oil + DMSO	0 mg/kg/day + 100 mg/kg/day
BPA5	BPA + Olive Oil + DMSO	5 mg/kg/day
BPA5Ti100	BPA + TiO_2_ NPs + Olive Oil + DMSO	5 mg/kg/day + 100 mg/kg/day
BPA50	BPA + Olive Oil + DMSO	50 mg/kg/day
BPA50Ti100	BPA + TiO_2_ NPs + Olive Oil + DMSO	50 mg/kg/day + 100 mg/kg/day

*n* = number of animals used in each group.

## Data Availability

The data presented in this study are available in this article.

## Supplementary Materials

The following are available online at https://www.mdpi.com/article/10.3390/foods11121696/s1. Figure S1: Rarefaction curve representing species richness; Figure S2: Changes at the genus level in both BPA-only exposure groups and in the groups receiving combined exposure to TiO2 NPs and BPA; Figure S3: Faecal titanium content is combined in the TiO_2_ NPs and BPA exposure groups and in the single-TiO_2_ NPs exposure groups; Table S1: Animal groupings and treatments; Table S2: Key parameters for GC-MS analysis (SCFAs); Table S3: Key parameters for GC-MS analysis (non-targeted metabolomics).
