# Supplementary material for: Intestinal Microecology of Mice Exposed to TiO2 Nanoparticles and Bisphenol A
Source: Foods. 2022 Jun 9;11(12):1696. doi: 10.3390/foods11121696 (PMC9222895; doi:10.3390/foods11121696)
Supplement: Supplementary file 1 [file foods-11-01696-s001.zip › foods-1715878-supplementary.pdf]

Supplementary information

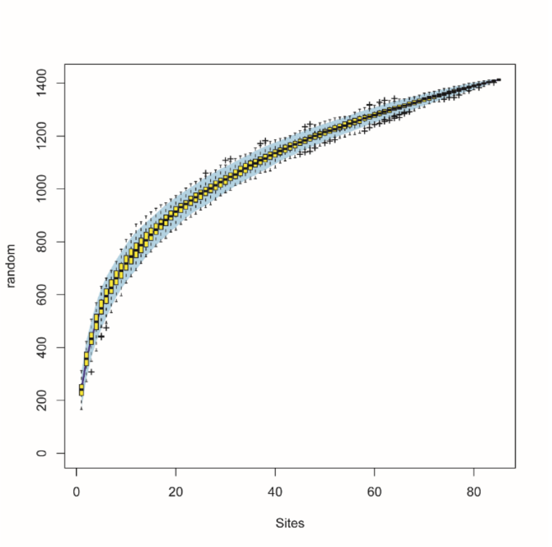

Figure S1 Rarefaction curve representing species richness. Data points represent mean OTU observed at a certain number of reads ± SEM.

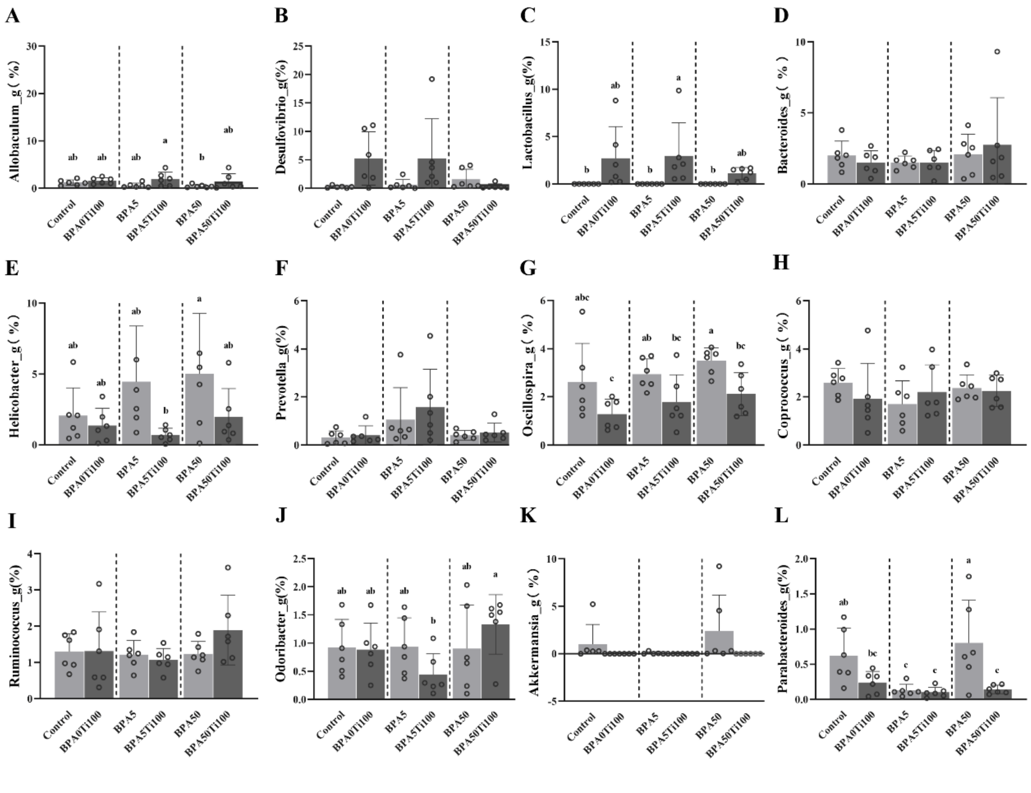

Figure S2 Changes at genus level in both BPA-only exposure groups and combined exposure of TiO_2 NPs and BPA groups. Data represent means of relative abundance of bacteria taxa ± SEM. The same letters represent no significant difference among groups ($p > 0.05$).

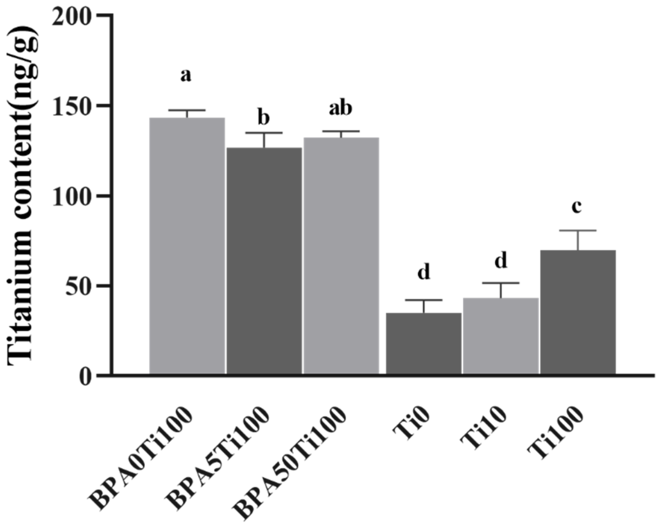

Figure S3 Fecal Titanium Content in combined of TiO_2 NPs and BPA exposure groups and single-TiO_2 NPs exposure groups. Data represent means ± SEM. The same letters represent no significant differenceamong groups ($p > 0.05$).

Table S1 Animal grouping and treatments.

**Groups, *N*=6**	**Treatment**	**Gavage dose and duration (13 weeks)**
Ti0	PBS	10mL/kg/day
Ti10	TiO_2 NPs + PBS	10mg/kg/day
Ti100	TiO_2 NPs + PBS	100 mg/kg/day

Table S2 Key parameters for GC-MS analysis (SCFAs).

	Parameters
Injection Volume	1μL
Front Inlet Mode	10:1
Carrier Gas	Helium
Column	DB-FFAP (30m ×0.25mm ×0.25μm)
Column Flow	1 mL/min
Oven Temperature Ramp	100 ℃ hold on 1min, raised to 160 ℃ at a rate of 5 ℃/min,raised to 250 ℃ at a rate of 80 ℃/min, hold on 6 min
Front Injection Temperature	260 ℃
Ion Source Temperature	230 ℃
Ionization Mode	EI
Electron Energy	70 eV
Scan mode	SIM

Table S3 Key parameters for GC-MS analysis (non-targeted metabolomics).

	Parameters
Injection Volume	1μL
Front Inlet Mode	No spilt

Carrier Gas	Helium
Column	HP-5 MS (60m × 0.32mm × 0.25μm)
Column Flow	1 mL/min
Oven Temperature Ramp	50 ℃ hold on 2 min, raised to 250 ℃ at a rate of 10 ℃/min, hold on 15 min, raised to 320 ℃ at arate of 10 ℃/min, hold on 10 min
Front Injection Temperature	250 ℃
Ion Source Temperature	230 ℃
Ionization Mode	EI
Electron Energy	70 eV
Scan range	50-700 m/z
